# New detection method of SARS-CoV-2 antibodies toward a point-of-care biosensor

**DOI:** 10.3389/fbioe.2023.1202126

**Published:** 2023-07-06

**Authors:** Janikua Nelson-Mora, Diana Rubio, Amairani Ventura-Martínez, Luis A. González, Diana Del-Rio, Yuli Aranda-López, Edgar Jiménez-Díaz, Diego Zamarrón-Hernández, Diana G. Ríos-López, Stephanie Aguirre, Yasab Ruiz-Hernandez, Aarón Cruz-Ramírez, Jonás S. Barjau, Miguel A. Jáurez, Jehú Lopez-Aparicio, Andrea Campa-Higareda, Tatiana Fiordelisio

**Affiliations:** ^1^ Unidad de Biología Molecular y Diagnóstico, Laboratorio Nacional de Soluciones Biomiméticas para Diagnóstico y Terapia LaNSBioDyT, Facultad de Ciencias, Universidad Nacional Autónoma de México, Mexico City, Mexico; ^2^ Laboratorio de Neuroendocrinología Comparada-LaNSBioDyT, Facultad de Ciencias, Universidad Nacional Autónoma de México, Mexico City, Mexico; ^3^ Unidad de Imagenología Cuantitativa, Laboratorio Nacional de Soluciones Biomiméticas para Diagnóstico y Terapia LaNSBioDyT, Facultad de Ciencias, Universidad Nacional Autónoma de México, Mexico City, Mexico

**Keywords:** fluorescence, immunodetection, microfluidic chip, COVID-19, magnetic beads

## Abstract

The outbreak of COVID-19, a disease caused by severe acute respiratory syndrome coronavirus 2 (SARS-CoV-2) infection, is regarded as the most severe of the documented coronavirus pandemics. The measurement and monitoring of SARS-CoV-2 antibody levels by serological tests are relevant for a better epidemiological and clinical understanding of COVID-19. The aim of this work was to design a method called the SARS-CoV-2 antibody detection method (SARS-CoV-2 AbDM) for fluorescence immunodetection of anti-SARS-CoV-2 IgG and IgM on both plate and microfluidic chip. For this purpose, a system with magnetic beads that immobilize the antigen (S protein and RBD) on its surface was used to determine the presence and quantity of antibodies in a sample in a single reaction. The SARS-CoV-2 AbDM led to several advantages in the performance of the tests, such as reduced cost, possibility of performing isolated or multiple samples, potential of multiplex detection, and capacity to detect whole blood samples without losing resolution. In addition, due to the microfluidic chip in conjunction with the motorized actuated platform, the time, sample quantity, and operator intervention during the process were reduced. All these advantages suggest that the SARS-CoV-2 AbDM has the potential to be developed as a PoC that can be used as a tool for seroprevalence monitoring, allowing a better understanding of the epidemiological and clinical characteristics of COVID-19 and contributing to more effective and ethical decision-making in strategies to fight against the COVID-19 pandemic.

## 1 Introduction

The current pandemic of coronavirus disease 19 (COVID-19), due to infection with severe acute respiratory syndrome coronavirus 2 (SARS-CoV-2), is considered the most severe of the documented coronavirus outbreaks and one of the most complex, multifaceted, and devastating challenges humanity has faced in the 21st century. Although the fatality rate is lower than that of other highly pathogenic beta coronaviruses, such as severe acute respiratory syndrome virus (SARS-CoV) and middle east respiratory syndrome virus (MERS-CoV), its spread has been such that the number of affected countries (228), cases (>617 million), and associated deaths (>6.5 millions), from its origin in December 2019 (Wu et al., 2020) to date, is overwhelmingly higher (Shi et al., 2020; Osuchowski et al., 2021; Worldometers.info, 2022).

Although SARS-CoV-2 and SARS-CoV have many similarities, present structural genomic and phenotypic differences influence pathogenesis (Mousavizadeh and Ghasemi, 2021; Osuchowski et al., 2021). These viruses share high genomic homology (79.6%) and, in general, have a high amino acid identity (>90%) in their structural proteins, except the spike protein (S), whose similarity is lower (76.7%–77.0%), especially in the S1 domain (64%) (Lu et al., 2020; Shi et al., 2020; Zhou et al., 2020; Hu et al., 2021). Both beta coronaviruses recognize angiotensin-converting enzyme 2 (ACE2) as its receptor.

S protein plays a crucial role in the infection process by mediating viral entry into cells through binding of the RBD in the S1 subunit to the ACE2 receptor and subsequently with membrane fusion through the S2 subunit (Harrison et al., 2020; Tai et al., 2020). Structural and biophysical evidence points to amino acid sequence changes in critical motifs of the S1/RBD and that SARS-CoV-2 binding through this domain to the ACE2 receptor is 10- to 20-fold more cognate than that of SARS-CoV RBD (Wrapp et al., 2020). This may influence the high transmissibility of SARS-CoV-2 and the fact that approximately 90% of the activity of neutralizing IgG antibodies is precisely directed toward RBD (Piccoli et al., 2020; Bachmann et al., 2021).

Likewise, the differences between SARS-CoV and SARS-CoV-2 RBD are sufficiently important that there is no cross binding of specific monoclonal antibodies (Wrapp et al., 2020). Consequently, it is pertinent to define S protein, particularly the RBD, in SARS-CoV-2 as both a therapeutic and diagnostic target (Shi et al., 2020; Tai et al., 2020; Wrapp et al., 2020; Zhao et al., 2020; Emeribe et al., 2022).

During viral infections, innate and adaptive immune responses are triggered (Abdulamir and Hafidh, 2020; Prompetchara et al., 2020; Sette and Crotty, 2021). In the particular case of SARS-CoV-2, it is reported that the virus is effective in evading the triggering of early innate immune responses (Sette and Crotty, 2021), delaying the adaptive response onset while the infection progresses (Cevik et al., 2020; Sette and Crotty, 2021). Multiple classes of immunoglobulins, especially IgM and IgG, are involved in the adaptive immune response. In COVID-19, the cumulative seroconversion rate increases rapidly during the first 2 weeks of the onset of SARS-CoV-2 infection, exceeding 50% (Zhao et al., 2020). The adaptive response begins during the ongoing infection (between 1 week and 2 weeks), with the increase of IgM levels until reaching a maximum (in 3 weeks) and then decreasing in an inverse relationship with the concomitant increase of IgG levels (3 weeks–7 weeks), whose affinity for the antigen and neutralization capacity is greater (Galipeau et al., 2020; Guo et al., 2020; Post et al., 2020; Sette and Crotty, 2021; Emeribe et al., 2022).

Notwithstanding these generalities and given the differences observed in the profile of the immune response in the general population (Emeribe et al., 2022), the measurement and monitoring of IgM and IgG levels by serological tests are relevant to characterize the responses, complement the diagnosis in patients with COVID-19 at different stages, and contribute to a better understanding of the pathogenesis of this disease (Azkur et al., 2020).

Given the global spread and prevalence of SARS-CoV-2, this virus is expected to become endemic. However, many elements of COVID-19 immunity are still unknown, including the duration of immunity, medium- and long-term immunologic effect of the vaccines used, and response to future vaccines developed (Galipeau et al., 2020; Goel et al., 2021; Huggett et al., 2021). The usefulness of serological assays in COVID-19 is, therefore, multiple and consequent in decision-making. From an epidemiological perspective, a serological assay could be used to identify the proportion of individuals exposed to the virus in various populations so that the evolution of disease incidence and the acquisition of social immunity can be closely monitored (Galipeau et al., 2020; Ravi et al., 2020).

Serological tests used for the detection of SARS-CoV-2-specific antibodies (mainly IgM and IgG) include the enzyme-linked immunosorbent assay (ELISA), chemiluminescent immunoassay (CLIA), and rapid diagnostic tests (RDTs) (Prazuck et al., 2020; Ravi et al., 2020). ELISA, considered the gold standard, is a quantitative test in which antibody titration can be performed; its basic process involves coating the plate with a known capture antigen that binds to the antibody present in the sample (usually serum or plasma), and these react with secondary antibodies bound to enzymes to produce a chromogenic reaction (Lin, 2015; Ravi et al., 2020). However, the protocol is relatively complex and long (2–8 h), of medium cost, and requires specialized personnel, instrumentation, and laboratories (Yüce et al., 2021).

The most common RDTs are those employing lateral flow chromatographic immunoassays (LFIAs) (Prazuck et al., 2020; Ravi et al., 2020). In this case, the treated sample migrates by capillary action and, if it contains viral antibodies, reacts with immobilized recognition antibodies conjugated with colloidal gold or colored latex particles. The result of these tests is binary (absence or presence). They are of low cost, require a small amount of sample (often a finger prick blood drop), simple and rapid (3–30 min), and usually consist of a portable point-of-care (PoC) device and paper analysis (Carrio et al., 2015; Koczula and Gallotta, 2016; Moreno et al., 2017; Kubina and Dziedzic, 2020; Ravi et al., 2020). However, in addition to being less specific and sensitive than ELISA, they do not quantify antibody titer (Yüce et al., 2021).

The COVID-19 pandemic caused an abrupt and unprecedented increase in the demand for antibody-based tests for healthcare or research purposes. This generated a resource-limited setting for tests and laboratory supplies, not just in managing COVID-19 patients but also for those with many other major medical conditions, with low-income and middle-income countries (LMICs) being the most affected (Bong et al., 2020). Thus, in response to the needs arising from this pandemic, which is expected to recur in the future, a call has been made to evaluate and create new detection methods that allow the constant monitoring of biomarkers of individual relevance not only for the health of the person but also for the population, as is the case of antibodies (Hallal et al., 2020; Vengesai et al., 2021; Emeribe et al., 2022).

PoCs, such as microfluidic chips, have become an important diagnostic tool in medicine. Their main advantage is to speed up diagnosis and allow the monitoring of diseases, mainly in non-urban areas or areas with limited access to medicine in LMICs, and are characterized by their low cost and high efficiency (Wieland et al., 2020). Therefore, the objective of this work is designing a method for the detection of IgG and IgM anti-SARS-COV-2 antibodies and implementing it in a microfluidic chip.

The method, hereinafter called the SARS-CoV-2 antibody detection method (SARS-CoV-2 AbDM), employs a magnetic bead system that immobilizes the antigen (S protein or RBD) on its surface and allows determining the presence and quantity of both antibodies in a serum or whole blood sample in a single reaction. Our working group has already implemented this technology for the detection of hypersensitivity pneumonitis antibodies (Fiordelisio et al., 2021), where it was observed that the use of magnetic beads, due to their small size and spherical geometry, allows for maximizing the number of immobilized biomolecules and increasing the sensitivity of the immunoassays besides reducing the reaction times and volumes of solutions and antibodies.

## 2 Materials and methods

### 2.1 Sample collection

The experimental protocol complies with all UNAM ethical guidelines for work in humans and was approved by the Commission of Academic Ethics and Scientific Responsibility of the Faculty of Sciences of UNAM (Folio: PI_2020_-2_003). All participating volunteers gave and signed informed consent.

Blood samples (*n* = 50) were obtained from adults between 21 and 86 years, without sex distinction, and residents of the metropolitan area of Mexico City, with 3–4 weeks of previous positive diagnosis of SARS-CoV-2 by RT-qPCR or who had been vaccinated for COVID-19. The inclusion criterion was that the sample had been previously determined as positive or negative by the ELISA method. Samples were identified as positive if they showed an absorbance with a cut-off greater than 0.4. Whole blood was obtained by venipuncture into collection tubes with a coagulation activator and separator gel (BDN-368159, BD Vacutainer, New Jersey, United States); after leaving them for 30 min at room temperature to clot, the samples were centrifuged at 1350 RCF for 15 min (5430-R, Eppendorf, Hamburg, Germany), and the resulting supernatant was collected as serum and stored at −70°C until used. For assays in which blood serum and whole blood samples were compared, these were obtained on the same day of the assay. Whole blood samples were collected in heparin tubes (BDN-367878, BD Vacutainer, New Jersey, United States).

### 2.2 SARS-CoV-2 antibody detection method

The SARS-CoV-2 AbDM is based on the one presented by Fiordelisio et al. (2021), where magnetic beads functionalized with an antigen react with a serum sample (with an unknown concentration of primary antibodies to be detected) and subsequently with a fluorescent conjugated secondary antibody to detect the emitted signal.

#### 2.2.1 Functionalization

Assays were performed with two different SARS-CoV-2 antigens: S protein and RBD. The protocol for coupling the antigen to the magnetic beads (Dynabeads™ Tosylactivated M-450, Invitrogen, Carlsbad, California, United States) followed the manufacturer’s instructions. These magnetic beads bind covalently to primary amino and sulfhydryl groups present in proteins such as RBD and S-protein.

For IgM and IgGS protein antibodies detection assays (C.V S-Protein Construct 6, LakePharma, California, United States), 240K magnetic beads were used per reaction, while for anti-RBD (S protein RBD (319-591-His10) LakePharma, 46438, Belmont, California, United States), 360K beads were used. To select the concentration of S protein and RBD, the theoretical antigen binding capacity of the beads established by the manufacturer was used as indicative and determined experimentally. A dilution curve containing at least one point with excess antigen was selected from these values. The tested antigen concentrations for the S protein were 0, 30, 60, 120, 240, 360, 480, and 600 ng/240K beads, and for RBD, it was 0, 240, 360, 360, 480, and 720 ng/360K beads.

To functionalize the magnetic beads, they were transferred to 500 μL of buffer 1 solution (B1; NaH_2_PO_4_ 0.1 M, Na_2_HPO_4_ 0.1 M pH = 8, S0751 and S0876, Sigma-Aldrich, Darmstadt, Germany) in 1.5 mL tubes (MCT-150-C, Axygen no pyrogenic and RNase/DNase-free, Corning, New York, United States), resuspended by 30 s vortexing. Beads were pooled using a magnet, the supernatant was discarded, and the antigen solution was subsequently added.

The total volume in each tube was 500 µL of functionalization solution (B1 added with antigen at corresponding concentration). The mixture was incubated for 24 h at room temperature (RT) under constant agitation in HulaMixer (HulaMixer Sample Mixer, Invitrogen, Carlsbad, United States). Subsequently, two washes were performed with buffer 2 solution (B2; Dulbecco’s PBS, 10 mM EDTA, supplemented with 0.2% BSA pH 7.4; D5652, E9884, and A2156, Sigma-Aldrich, Darmstadt, Germany).

To block the remaining tosyl groups, 500 µL of buffer 3 solution (B3; tris base 0.2 M supplemented with 1% BSA pH = 8.5; T1503 Sigma-Aldrich, Darmstadt, Germany) was added. The mixture was incubated for 16 h in HulaMixer at 37°C in a benchtop incubator (New Brunswick™ Innova^®^ 40/40R, Eppendorf, Hamburg, Germany). Two washes were performed with B2 and finally resuspended in buffer 4 (B4: Dulbecco’s PBS, D5652, Sigma-Aldrich, Darmstadt, Germany) to seed 50 µL a in 96-well plate (previously blocked with 0.05% BSA) and in buffer 4T (B4T: Dulbecco’s PBS, supplemented with 0.01% Tween 20; P1379, Sigma-Aldrich, Darmstadt, Germany) to load 15 µL on the microfluidic chip, at the appropriate concentration of beads according to the antigen. In this way, they were ready for the uptake of anti-SARS-COV-2 IgG and IgM antibodies in the serum sample and their subsequent detection with the secondary antibody.

Serum samples with the highest ELISA-confirmed titer (8100) and a secondary antibody saturating condition were used to standardize the functionalization parameters. To verify whether there was a non-specific binding of serum or secondary antibody, beads functionalized without antigen, i.e., without RBD (woRBD) or S (woS), were used as controls, using B1 added with 1% BSA as the functionalization solution. The washing and blocking processes were performed under the same conditions as the antigen-functionalized beads.

#### 2.2.2 Detection reaction

For detection of the anti-SARS-CoV-2 antibodies present in the serum samples, anti-IgG (αHIgG-647) conjugated with Alexa Fluor^®^ 647 AffiniPure Donkey Anti-Human IgG H + L (RRID: AB_2340578; Jackson ImmunoResearch Labs Cat# 709-605-149, 1.25 mg/mL, West Grove, United States) and anti-IgM (αHIgM-488) conjugated to Alexa Fluor^®^ 488 AffiniPure Donkey Anti-Human IgM Fc Fragment specific (RRID: AB_2340564; Jackson InmunoResearch Labs Cat# 709-545-073, 1.33 mg/mL, West Grove, United States) were used.

For antibody recognition assays, a dose–response curve was performed to determine the saturation point for RBD antigen (0.018, 0.036, 0.054, 0.072, and 0.09 μg/μL) and S protein antigen (0.012, 0.024, 0.036, 0.048, and 0.06 μg/μL) recognition in a 96-well plate (Costar 3915, Corning, Maine, United States). This parameter was also determined for the microfluidic chip reaction (0.012, 0.024, 0.036, 0.048, 0.06, 0.08, and 0.093 μg/μL) with S protein as antigen.

A final volume of 50 µL of secondary antibody solution in B4 was used for IgG and IgM plate recognition, and a final volume of 15 µL prepared with B4T was used for the microfluidic chip. For the plate detection reactions, incubation was performed at RT with shaking (250 rpm) for 2 h; finally, three washes were performed with B4 and resuspended in 50 µL of the same buffer. On the other hand, in the microfluidic chip, the incubation was 45 min at RT, with agitation by transverse magnet movement (TMM) (Fiordelisio et al., 2021). Subsequently, a wash was performed by transiting the beads through B4T and resuspending them in the same buffer for reading. For both platforms, a serum sample with ELISA titer of 8100 was used.

#### 2.2.3 Measurement of anti-RBD and anti-S antibodies

The optimal amount of serum sample was determined by a dilution curve. Serum samples were prepared for both antigens according to the reaction platform: 50 µL of a B4-diluted serum sample (1:20, 1:10, 1:5, and 3:10) was used for the plate reactions, while 15 µL of the B4T-diluted serum sample (1:6, 1:3, and 2:3) was used for the chip.

For the measurement of antiSARS-CoV-2 antibodies on a plate, the serum sample solution was added to the functionalized beads and incubated at RT under agitation (250 rpm, 2 h). Then, two washes were performed (50 μL B4, 250 rpm, RT, 5 min). On the chip, incubation was performed for 45 min at RT with TMM agitation; a wash was performed by transiting the beads through B4T. In both cases, the detection reaction with the secondary antibody was performed as described previously.

#### 2.2.4 Fluorescent detection analysis

Upon completion of the detection reaction with the secondary antibodies, the plates and microfluidic chips were analyzed with a Cytation 5 multi-mode cell imager (CYT5MW BioTek, Winooski, Vermont, United States). Three representative images were captured at ×20 magnification using a CY5 filter cube (EX/EM 628/685) for IgG and a GFP filter cube (EX/EM 469/525) for IgM, and the following acquisition parameters were set: led intensity of 1, gain of 9, and integration of 100 ms (RBD and microfluidic chip) or 150 ms (S protein). The images were saved in tiff format and analyzed with *ad hoc* software designed to obtain the average fluorescence intensity and the standard deviation associated with each of the images obtained.

The designed software determines the fluorescence intensity per sample by calculating the fluorescence intensity at three different levels: bead, image, and sample. At the bead level, a Laplacian Gaussian filter (LoG filter) was used for segmentation and detection of beads in the images. After precise spatial localization, the fluorescence intensity of each bead was calculated by averaging the values of the set of pixels that constitute it. The minimum and maximum intensity levels, the number of identified beads, and the average fluorescence intensity per image were obtained. In addition to the average, two additional values were calculated: the 75th percentile and the sum of all N values above the 25th percentile divided by N (the weighted sum). Finally, the fluorescence intensity values of all images related to a single sample were used to calculate the same values (average fluorescence intensity, 75th percentile average, and weighted sum). In all cases, the average and associated standard deviation were calculated as well as the data used for the weighted sum.

To obtain a better resolution of the magnetic beads, the images presented in this work were obtained with an upright confocal microscope (TCS-SP8, LEICA, Wetzlar, Germany). For this purpose, an aliquot (10 µL) was placed on a slide, taking care to arrange the beads in a single focal plane using a magnet, and a slide was placed on the top. The magnetic beads were excited with a laser at 488 nm (IgM) or 638 nm (IgG). Emission was detected with a hybrid detector and collected at 493–570 nm and 646–708 nm, respectively. Samples were observed with a ×40/0.80 objective. Three photos of each well were taken and exported in tiff format for analysis.

#### 2.2.5 Microfluidic chip

**FIGURE 1:**
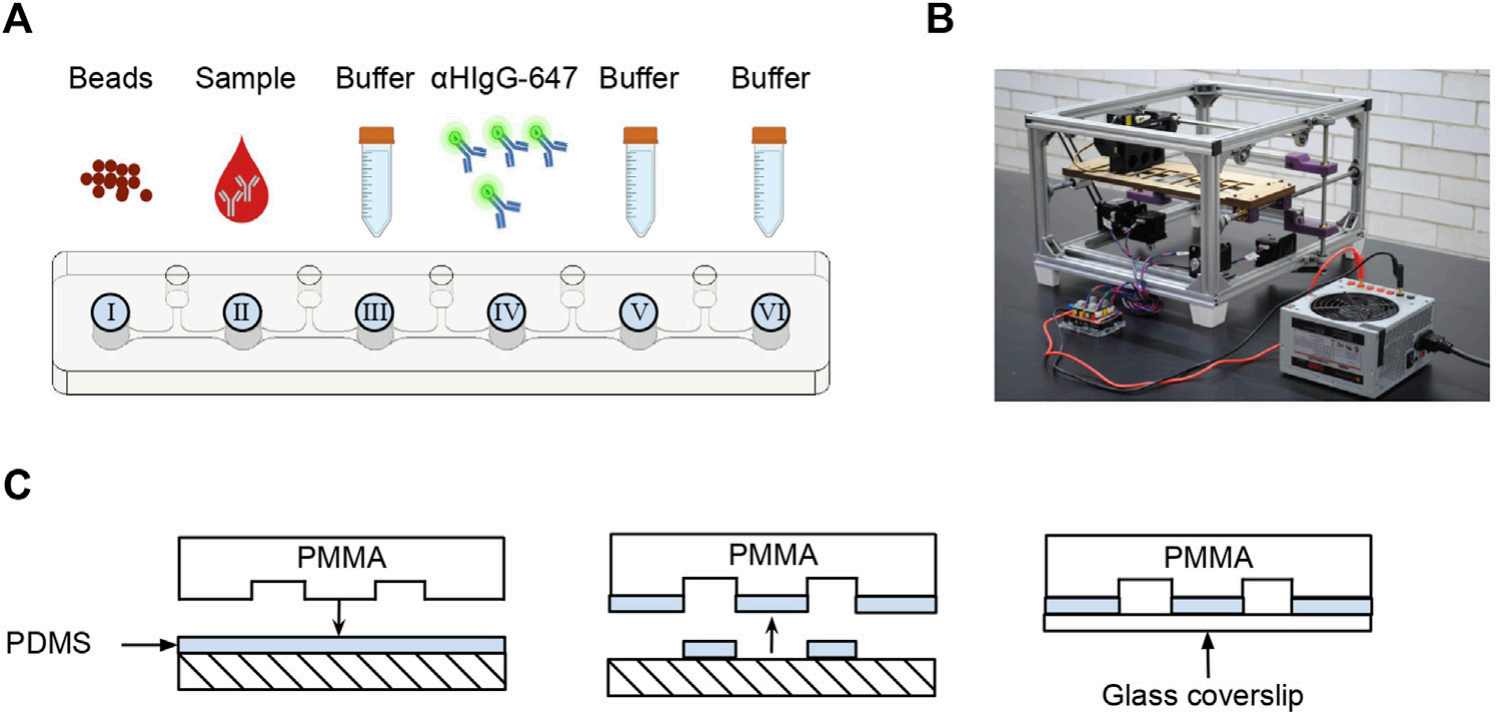
Microfluidic chip for SARS-CoV-2 AbDM. **(A)** Schema of the sequence of microchannel and the contents of each well. **(B)** Photograph of the automated platform used to move the magnetic beads in the microfluidic chip. **(C)** Sealing of the microfluidic chip. The microstructured PMMA is coated in uncured PDMS and then sealed with a coverslip.

The SARS-CoV-2 AbDM was implemented on a microfluidic chip making use of an automated platform, both designed by Fiordelisio et al. (2021). The chip consists of a series of six wells (I–VI) with different reagents connected by microchannels with mineral oil (M5904, Sigma-Aldrich, Steinheim, Germany) (Figure 1A) to prevent mixing of the different solutions. The chip is placed on the automated platform (Figure 1B) whose magnets move the functionalized beads along the series of wells where the different reactions and washes are carried out keeping them in agitation, during each reaction process.

As an alternative to the process of hermetically sealing the chip with polymethyl methacrylate (PMMA) and absolute ethanol (Fiordelisio et al., 2021), adhesion was performed with a 60 × 24 mm glass coverslip (2980-246, Corning, New York, United States) and polydimethylsiloxane (PDMS; SYLGARD™ 184 Silicone Elastomer Base and SYLGARD™ 184 Silicone Elastomer Curing Agent Kit, DOW, Michigan, United States) (Figure 1C) by thermal polymerization (60°C, 1 h) in an incubator (SI-950 Ultraviolet Incubator, Analytik Jena, California, United States).

Chip loading consisted of filling each well with the solutions in the following order: I, functionalized magnetic beads; II, serum sample solution; III, B4T as a wash solution; IV, secondary antibody solution; V, B4T for the wash; and VI, B4T for readout. After filling all wells, to avoid well contamination, a clean glass coverslip was placed on top and mounted on the platform for the execution of the programmed routine.

### 2.3 ELISA

For the detection of anti-S IgG/IgM and anti-RBD IgG/IgM, a screening ELISA was performed to determine positive and negative serum samples, and, subsequently, an ELISA for titer determination was performed for positive serum samples (Amanat et al., 2020; Martínez-Barnetche et al., 2022). In both protocols, a 96-well high-affinity plate (82.1581.200, SARSTEDT, Nümbrecht, Germany) was coated with S protein (C.V.S Protein RBD Construct 6, LakePharma, California, United States) or with RBD (S Protein, RBD (319-591)-His10, LakePharma, California, United States) and incubated at 4°C overnight. Subsequently, three washes with PBS containing 0.1% Tween 20 (PBS-T; Dulbecco’s PBS; Tween 20; D5652 and P1379, Sigma-Aldrich, Darmstadt, Germany) and a block with PBS-T supplemented with 3% milk (Svelty low fat, Nestlé S.A de C.V, CDMX, Mexico) were performed for 3 h at RT.

Briefly, serum samples were pre-diluted 1:5 in PBS for subsequent dilutions in PBS-T supplemented with 1% milk in a 96-well low affinity plate (Costar 9017, Corning, Maine, United States), both for screening ELISA (1:50) and for ELISA for titer determination of IgG (1:100, 1:300, 1:900, 1:2700, and 1:8100) and IgM (1:100, 1:200, 1:400, 1:800, and 1:1600) antibodies. These dilutions were transferred (50 µL) to the plate coated with S protein (80 ng/well) or RBD (100 ng/well), incubated (2 h at RT), and then extensively washed with PBS-T.

Next, goat anti-human IgG:HRP (RRID:AB_619881, Cat# 204005, Bio-Rad, California, United States; αHIgG-HRP) and goat F (ab')2 anti-human IgM-HRP (MBS675102, MyBioSource, California, United States; αHIgM-HRP) secondary antibody solution was added 1:3000 in PBS-T supplemented with 1% milk (50 µL) and incubated (50 min at RT). Next, three washes were performed with PBS T and incubated in 100 µL OPD solution (0.402 mg/mL OPD; o-phenylenediamine dihydrochloride: P1526, Sigma-Aldrich, Missouri, United States; and 0.02 mg/mL H_2_O_2_; 10 min, TA and in the dark), and 3N HCl was used as a stop solution (50 µL). Finally, OD_492nm_ was determined on the Cytation 5 reader.

### 2.4 Statistical analysis

One-way ANOVA with *post hoc* Tukey or Z test was used for the differences between the means of the assays. An α of 0.05 was set for significant differences. All statistical analyses were performed with GraphPad Prism software (v. 9, GraphPad Software, Greenville, Columbia, United States).

To determine the limit of the detection (LoD) of the SARS-CoV-2 AbDM, the definition by Mac Dougall and co. (1980) was used: the detection limit signal of an analytical method should be greater than three times the standard deviation of the blank measurement. In this case, the control functionalized without antigen was used as blank. This LoD is presented as the lower limit of the detection range.

ROC analysis was performed in PSPP statistics separately for RBD and S protein (v. 1.4, GNU Software, Boston, Massachusetts, United States).

## 3 Results

### 3.1 SARS-CoV-2 AbDM standardization

To optimize the SARS-CoV-2 AbDM, before its validation and application on microfluidic chips, several parameters were standardized using the RBD or S protein as antigen. These parameters included the optimal amount of antigen to functionalize the magnetic beads, the amount of secondary antibody to obtain the highest possible detection of anti-SARS-CoV-2 antibodies in the sample, and the ratio of serum sample dilution.

#### 3.1.1 RBD as antigen

**FIGURE 2:**
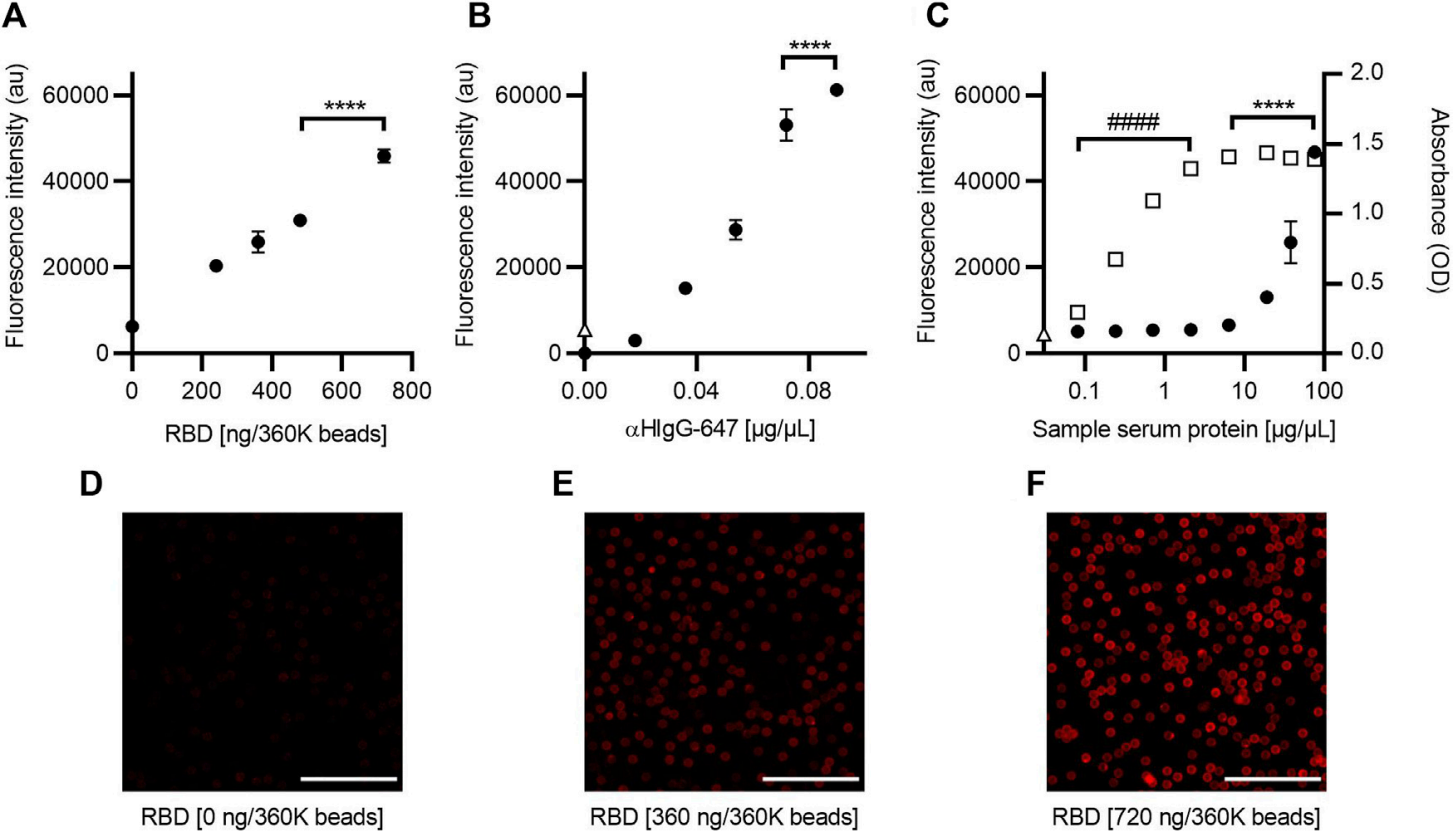
Optimization of the SARS-CoV-2 AbDM for IgG-RBD detection. **(A)** Plot of the fluorescence intensity obtained for different concentrations of RBD, used in the functionalization of the beads (360K beads), and **(B)** for different concentrations of αHIgG-647. **(C)** Comparative plot of the fluorescence and absorbance measurement obtained for IgG-RBD with the SARS-CoV-2 AbDM and with ELISA, respectively. Plotted (●) is the result of SARS-CoV-2 AbDM and (◻) is the values obtained by ELISA. In **(B)** and **(C)**, Δ represents the fluorescence value of the control beads functionalized without RBD (woRBD). In all plots, symbols represent the mean of three replicates ± SD. Statistically significant differences are shown with **** (*p* < 0.0001) for SARS-CoV-2 AbDM and with #### (*p* < 0.0001) for ELISA. **(D–F)** Representative confocal microscopy images of **(A)**. Scales: 50 μm.

A direct relationship between fluorescence intensity and the amount of RBD was observed when determining the optimal amount of RBD antigen to functionalize the beads (Figure 2A). Based on these results, it was decided to use 720 ng/360K beads, as this condition showed a 1.5-fold increase in fluorescence intensity compared to the lowest concentration (ANOVA, *F* = 271.4, *p* < 0.0001, and Tukey, *p* < 0.0001) and homogeneous labeling in the bead population (Figures 2D–F).

In the determination of the optimal concentration of secondary antibody (αHIgG-647) for the anti-RBD antibodies detection carried out in the SARS-CoV-2 AbDM plate-based method, a tendency of saturation of the fluorescence signal was observed after 0.072 μg/μL, with the difference being significant at 0.09 μg/μL (ANOVA *F* = 653.4, *p* < 0.0001, and Tukey *p* < 0.001) (Figure 2B). Therefore, the concentration of αHIgG-64 to be used was determined to be 0.09 μg/μL.

The fluorescence intensity of a series of dilutions (0.08, 0.24, 0.71, 2.13, 6.40, 19.20, 38.41, and 76.81 μg/μL total protein) of an ELISA-confirmed serum titer of 8100 was analyzed to determine the optimal sample concentration for detection and to compare the measurement of IgG anti-RBD antibodies between the ELISA and the SARS-CoV-2 AbDM plate-based method. In all cases, the volume of serum added to the reaction was 2.5 µL (Figure 2C). It was observed that the SARS-CoV-2 AbDM allows discriminating differences from 19.20 μg/μL (ANOVA, *F* = 209, *p* < 0.0001, and Tukey, *p* < 0.0001) and that the saturation point is not reached with the concentrations evaluated. On the other hand, with the ELISA method, the positive sample can be distinguished from 0.08 μg/μL and saturates from 6.40 μg/μL (ANOVA, *F* = 993, *p* < 0.0001, and Tukey, *p* > 0.05).

#### 3.1.2 S protein as antigen

**FIGURE 3:**
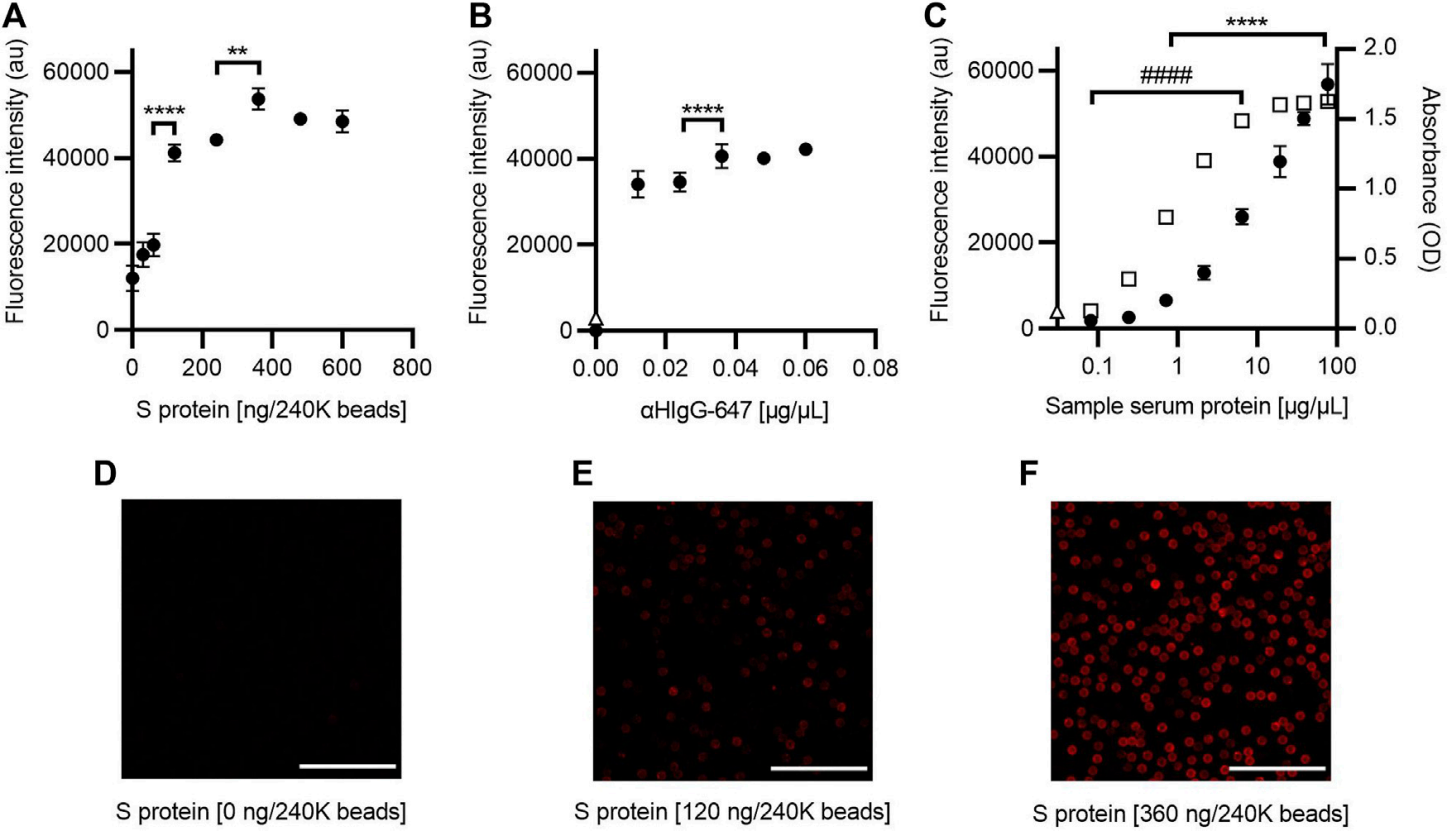
Optimization of the SARS-CoV-2 AbDM for IgG-S detection. **(A)** Plot of the fluorescence intensity obtained for beads (240K beads) functionalized with different concentrations of S protein and **(B)** for different concentrations of αHIgG-647. **(C)** Comparison of the fluorescence and absorbance measured for IgG-S using the SARS-CoV-2 AbDM and ELISA, respectively. Plot shows (●) the SARS-CoV-2 AbDM results and (◻) the ELISA values. In **(B)** and **(C)**, Δ represents the fluorescence intensity of the control beads functionalized without S protein (woS). In all plots, symbols represent the mean of three replicates ± SD. Statistically significant differences are shown with ** (*p* < 0.01) and **** (*p* < 0.0001) for SARS-CoV-2 AbDM and with ####, (*p* < 0.0001) for ELISA. **(D–F)** Representative confocal microscopy images of **(A)**. Scales: 50 μm.

The same assay strategy as for the RBD was used to standardize the SARS-CoV-2 AbDM using S protein as antigen. The saturation concentration to functionalize was estimated at 360 ng/240K beads (ANOVA, *F* = 137.3, *p* < 0.0001, and Tukey, *p* < 0.001), and adequate labeling is observed (Figure 3A, Figures 3D–F). Therefore, this value was selected for the other assays. As for the concentration of αHIgG-647 for plate detection (Figure 3B), 0.036 μg/μL was chosen because, from this condition, it does not increase fluorescence significantly.

Comparison of IgG-S measurement by ELISA and the SARS-CoV-2 AbDM plate-based method (Figure 3C) was also evaluated in the same way as for IgG-RBD; it was observed that, in this case, the SARS-CoV-2 AbDM discriminates from 0.71 μg/μL (ANOVA, *F* = 279, *p* < 0.0001, and Tukey, *p* < 0.05) and does not reach saturation in the conditions evaluated. On the other hand, the ELISA discriminates at 0.08 μg/μL, and the signal saturates at 19.20 μg/μL (ANOVA, *F* = 4084, *p* < 0.0001, and Tukey, *p* > 0.05).

### 3.2 Validation of the SARS-CoV-2 AbDM

**FIGURE 4:**
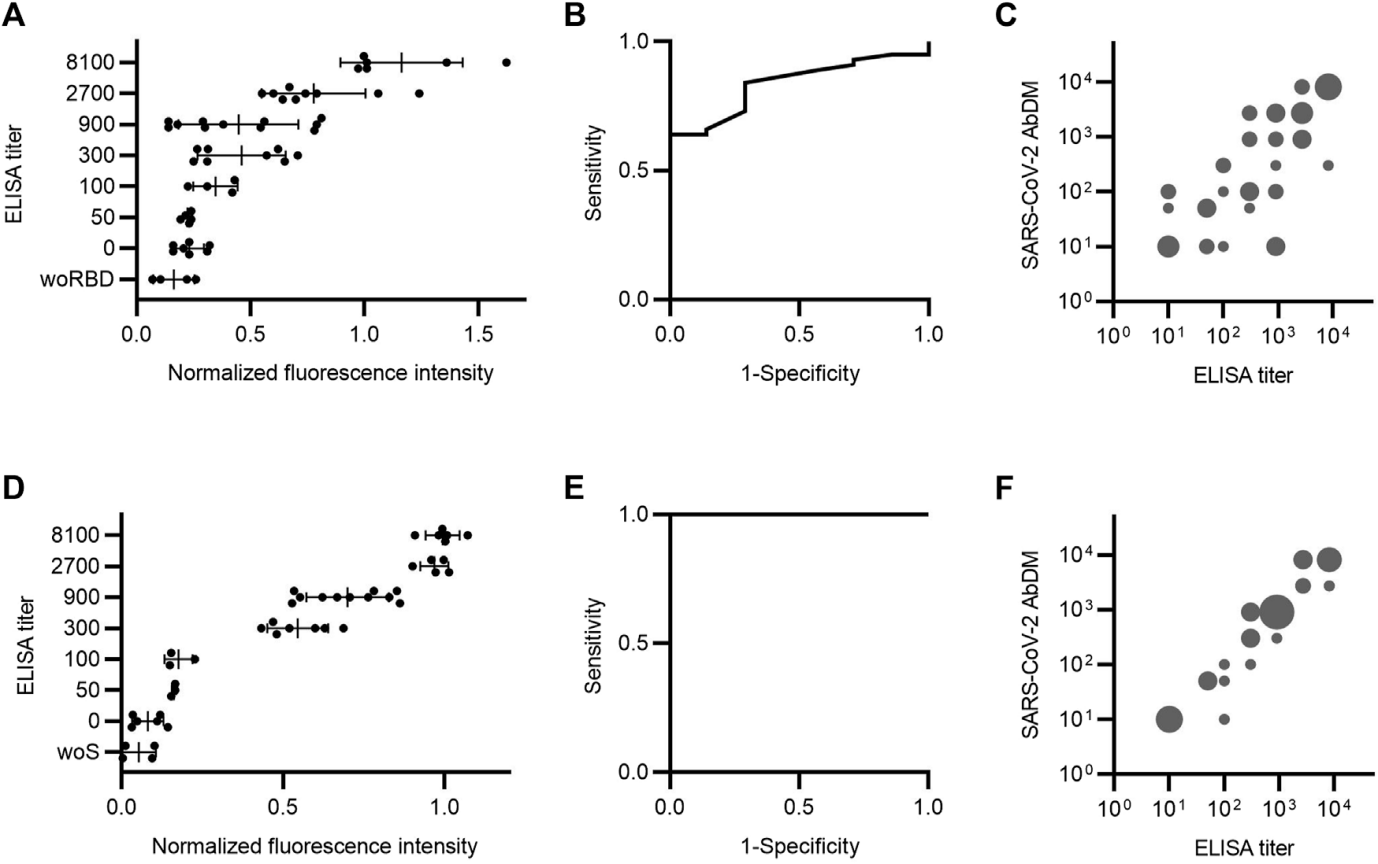
Serum antibody detection comparison between SARS-CoV-2 AbDM and ELISA. Samples were analyzed by both methods to detect **(A–C)** IgG-RBD (*n* = 50) and **(D–F)** IgG-S (*n* = 31). **(A** and **D)** ELISA titer versus SARS-CoV-2 AbDM normalized fluorescence intensity. The mean (●) of the SARS-CoV-2 AbDM result for each sample and the mean and deviation of each ELISA titer population are shown. woRBD and woS correspond to control beads functionalized without antigen. **(B** and **E)** Receiver operating characteristic (ROC) curve for SARS-CoV-2 AbDM, with an area under the curve of 0.84 for IgG-RBD and 1 for IgG-S. **(C** and **F)** SARS-CoV-2 AbDM titer versus ELISA titer. Cut-off points of the normalized fluorescence intensity were determined for each titer using ROC analysis. The size of the bubble is proportional to the number of incidences (1–10) in each range. All negative cases were plotted with the value of 10, as the scale is logarithmic.

To validate the SARS-CoV-2 AbDM, a selection of an equal number of serum samples from the different ELISA titer groups was tested for IgG-RBD (*n* = 50), and a different selection of samples was tested for IgG-S (*n* = 31) and compared with the ELISA. Each serum sample was determined in duplicate (four plates for RBD and three for S protein). In order to compare the results of each set of experiments, the values obtained were normalized, in each case, to the average value of the fluorescence intensity of the samples with the highest titer (8100). The normalized fluorescence intensity was compared against ELISA titers (Figures 4A, D). For both antigens, an increasing trend in the average normalized fluorescence intensity is observed in accordance with the increase in the ELISA titer. In addition, the dispersion in the populations of each titer is smaller in the detection of IgG-S (Figures 4D–F).

A receiver operating characteristic (ROC) curve was executed to assess the area under the curve (AUC) for the normalized fluorescence intensity of SARS-CoV-2 AbDM as a detection tool for IgG-RBD and IgG-S (Figures 4B, E). The AUC for RBD was 0.84, and the cutoff point of 0.23 is related to the highest specificity (71%) and sensitivity (84%) for the SARS-CoV-2 AbDM variable. On the other hand, the AUC for S protein was 1, and the cutoff point of 0.15 is related to the highest specificity (100%) and sensitivity (100%) for the SARS-CoV-2 AbDM variable.

To make the comparison between the two methods more direct, the cutoff points for each titer were determined from the normalized fluorescence intensity values. For this purpose, a ROC analysis was performed by changing the state variable for each titer, and the cut-off point at which sensitivity and specificity were optimal was extracted. Thus, the simile of the ELISA titer applied to the SARS-CoV-2 AbDM was obtained. To compare both methods, a bubble plot was constructed with the values obtained with SARS-CoV-2 AbDM against the ELISA titers represented in groups with incidences equivalent to the bubble size (Figures 4C, F). It was thus observed that there is a greater coincidence in the titers obtained by both methods in the detection of IgG-S, especially in titers greater than 900.

### 3.3 Detection in whole blood

**FIGURE 5:**
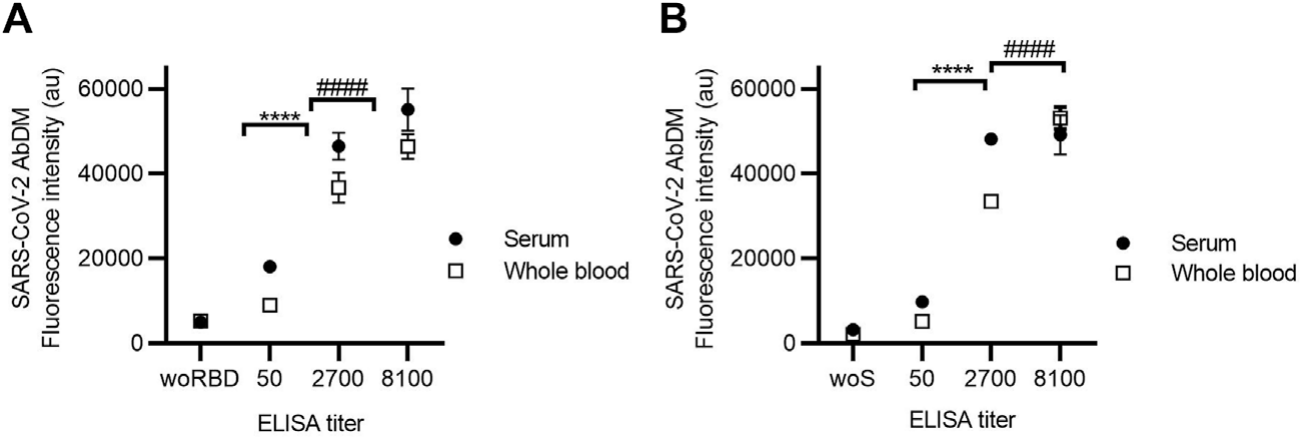
Detection of **(A)** IgG-RBD and **(B)** IgG-S in serum and whole blood using SARS-CoV-2 AbDM in ELISA-titrated samples. Mean of three replicates ± SD of (●) serum and (◻) whole blood fluorescence intensity values are plotted. woRBD and woS correspond to control beads functionalized without antigen and reacted with the highest titered sample. Statistically significant differences are shown with **** (*p* < 0.0001) for serum and with #### (*p* < 0.0001) for whole blood.

The process of obtaining serum from a blood sample, as indicated in the methods, requires at least 45 min, special equipment, and tubing, so it is possible to facilitate the detection process if the sample used is whole blood instead of serum. Therefore, the ability of the SARS-CoV-2 AbDM to detect IgG-RBD and IgG-S using whole blood was evaluated. For this purpose, IgG detection with SARS-CoV-2 AbDM was performed on both antigens in serum and whole blood samples (Figure 5), with ELISA titers determined in serum (50, 2700, and 8100). As shown in Figure 5, it is possible to measure the presence of antibodies in whole blood as well as in serum, showing significant differences in fluorescence intensity with respect to the controls (an increase in signal of 0.71 fold for RBD and 1.40 fold for S protein), both for the detection of IgG-RBD (Z test, Z = 4.7, *p* < 0.00001) and IgG-S (Z test, Z = 5.6, *p* < 0.00001), and it was possible to distinguish all the titers (RBD: ANOVA, *F* = 155.6, *p* < 0.0001; S: ANOVA, *F* = 265, *p* < 0.0001a 900).

### 3.4 Co-detection of IgG and IgM

**FIGURE 6:**
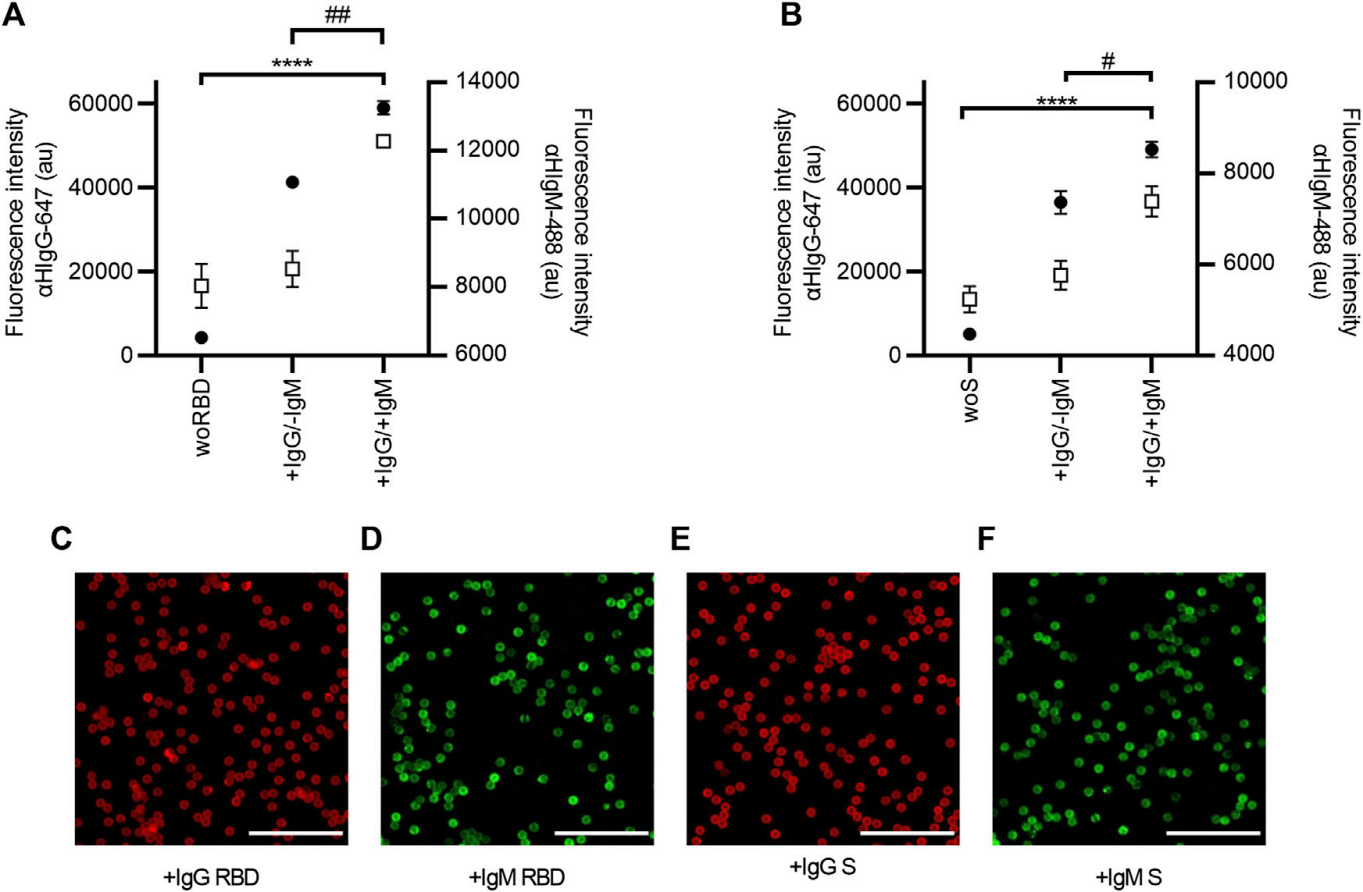
Co-detection of IgG and IgM in serum samples with SARS-CoV-2 AbDM, using **(A)** RBD and **(B)** S protein as antigen. Mean of three replicates ± SD of (●) IgG (αHIgG-647) and (◻) IgM (αHIgM-488) fluorescence intensity values are plotted. Two serum samples previously analyzed by ELISA were tested: a serum sample positive only for IgG (+IgG/-IgM) and a serum sample positive for both antibodies (+IgG/+IgM); woRBD and woS correspond to control beads functionalized without antigen and reacted with the +IgG/+IgM sample. Statistically significant differences by ANOVA are shown for IgG **** (*p* < 0.0001) and for IgM ## (*p* < 0.001) and # (*p* < 0.01). **(C–F)** Representative confocal microscopy images of IgG and IgM detection with both antigens. Scales: 50 μm.

The ability of SARS-CoV-2 AbDM to co-detect IgG and IgM of both antigens was evaluated in two serum samples, one positive only for IgG (+IgG/-IgM) and the other positive for both immunoglobulins (+IgG/+IgM), determined by ELISA (Figure 6). For this, the results obtained with SARS-CoV-2 AbDM were consistent because in the fluorescence intensity of the +IgG/-IgM sample in both antigens, significant differences were only observed for IgG (RBD: ANOVA, *F* = 946.1, *p* < 0.0001; Tukey, *p* < 0.0001; S: ANOVA, *F* = 310.9, *p* < 0.0001; Tukey, *p* < 0.001) with respect to the control, while for the +IgG/+IgM sample, it was significant for both IgG (RBD: ANOVA, *F* = 946.1, *p* < 0.0001; Tukey, *p* < 0.001; S: ANOVA *F* = 310.9, *p* < 0.0001; Tukey, *p* < 0.001) and for IgM (RBD: ANOVA, *F* = 31.23, *p* < 0.001; Tukey, *p* < 0.0001; S: ANOVA, *F* = 43.02, *p* < 0.001; Tukey, *p* < 0.0001).

### 3.5 Microfluidic chip implementation

**FIGURE 7:**
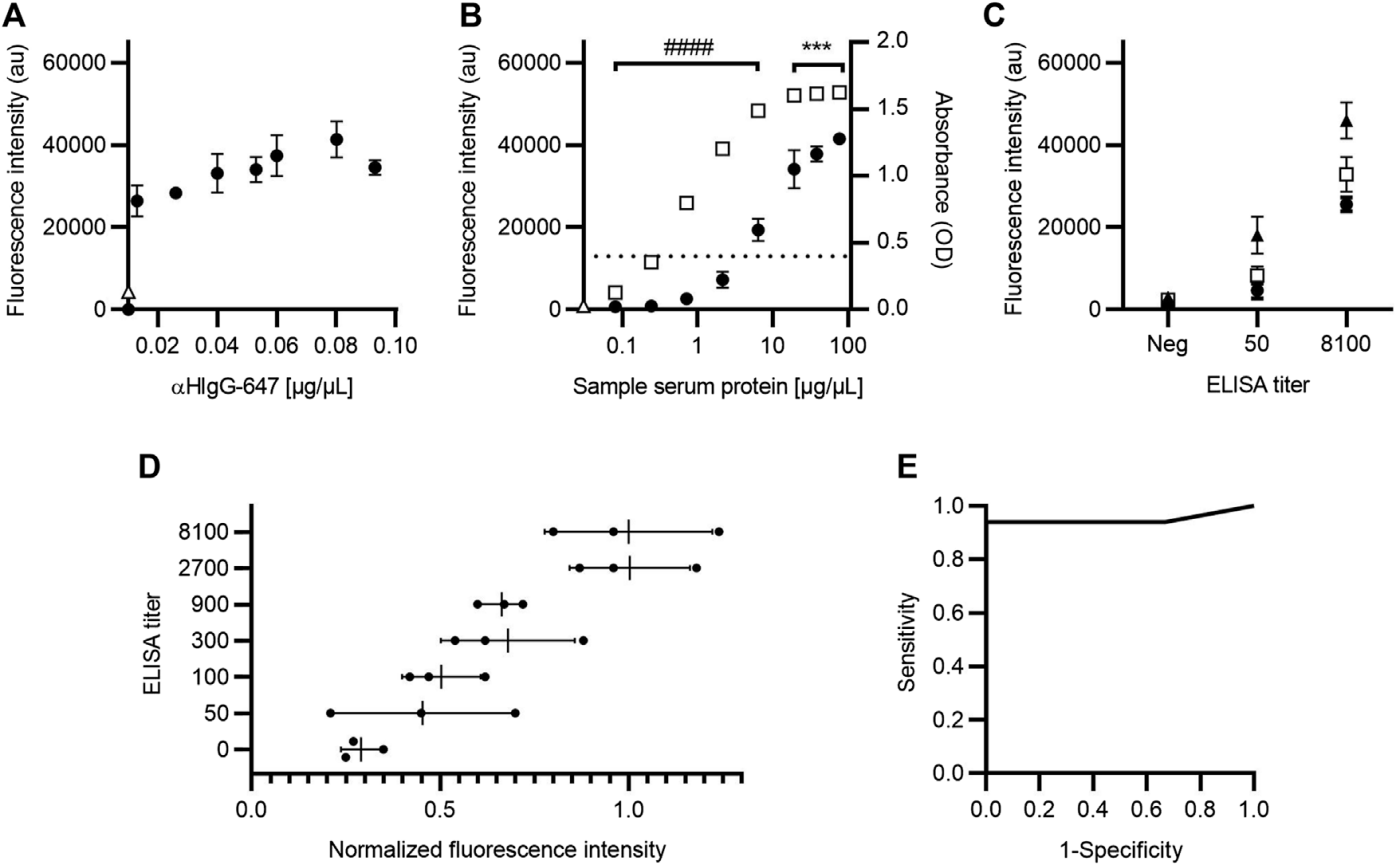
Optimization of IgG-S detection with SARS-CoV-2 AbDM for on-chip microfluidic implementation. **(A)** Fluorescence intensity plot for different secondary antibody (αHIgG-647) concentrations on-chip. **(B)** Comparison of fluorescence and absorbance measurements for IgG-S with SARS-CoV-2 AbDM on-chip and with ELISA, respectively. The SARS-CoV-2 AbDM results are shown with (●), and the ELISA values are shown with (◻). In **(A)** and **(B)**, (Δ) represents the fluorescence intensity of the control beads functionalized without S protein (woS). **(C)** Determination of serum sample dilution ratio. Fluorescence intensity of samples with different ELISA titers (negative; 50; 8100) are shown with (●) for 1:6, with (◻) for 1:3, and with (▲) for 2:3. **(D)** ELISA titer versus normalized fluorescence intensity of SARS-CoV-2 AbDM on-chip. The mean (●) of the SARS-CoV-2 AbDM result on chip for each sample (*n* = 21) and the mean and deviation of each ELISA titer population are shown. **(E)** ROC curve for SARS-CoV-2 AbDM on chip, with an area under the curve of 0.94 for IgG-S. Symbols represent the mean of three **(A–C)** or two **(D)** replicates ± SD. Statistically significant differences are shown with *** (*p* < 0.001) for SARS-CoV-2 AbDM and with #### (*p* < 0.0001) for ELISA.

The next step in the development of the SARS-CoV-2 AbDM was its implementation on a microfluidic chip for the detection of IgG-S toward a PoC device. For this purpose, it was necessary to optimize the secondary antibody concentration parameters and the serum sample dilution ratio; it is worth mentioning that the established functionalization parameters were maintained as this process is independent of the device. Thus, for the determination of αHIgG-647 concentration for on-chip detection (Figure 7A), 0.08 μg/μL was chosen, as, from this condition, it does not increase fluorescence significantly.

Regarding the comparison of IgG-S measurement between ELISA and on-chip SARS-CoV-2 AbDM (Figure 7B), this was performed in the same way as described for the plate and considering the concentration of αHIgG-647 established for the chip; consistent results were obtained with those presented for IgG-S on the plate (Figure 3C), resolving in SARS-CoV-2 AbDM on the chip from 2.13 μg/μL (ANOVA F = 139, *p* < 0.0001, and Tukey *p* < 0.0001) without reaching the saturation point at the concentrations used. Likewise, to evaluate the sample ratio to be used in the on-chip SARS-CoV-2 AbDM, serum samples of different ELISA titers (negative, 50, and 8100) diluted at 1:6, 1:3, and 2:3 ratios were analyzed (Figure 7C). We established 2:3 as the sample dilution ratio to be used in the chip as it presents the maximum fluorescence in both positive samples. In addition, in this ratio, the sample with the lowest titer (50) is significantly different from the negative one (ANOVA, *F* = 105.1, *p* < 0.0001; Tukey, *p* < 0.01).

As in the SARS-CoV-2 plate-based AbDM, the chip method was validated by analyzing serum samples for IgG-S detection (*n* = 21) and compared with ELISA. Each sample was determined in duplicate, the results were normalized to the average fluorescence intensity of 8100 titer samples, and the normalized fluorescence intensity was compared against ELISA titers (Figure 7D). As in the plate validation, an increasing trend in the average fluorescence intensity was observed as the ELISA titer increased. A ROC curve was executed for the fluorescence intensity of IgG-S, with SARS-CoV-2 AbDM on-chip as a detection tool (Figure 7E). The AUC was 0.94, and the cutoff point of 0.40 is related to the highest specificity (100%) and sensitivity (94%) for the SARS-CoV-2 AbDM variable.

## 4 Discussion

In order to design a method for the detection of anti-SARS-COV-2 IgG and IgM antibodies in human samples, we present the SARS-CoV-2 AbDM, which was implemented both in a multiwell plate and in a microfluidic chip. This method is based on fluorescence immunodetection of such immunoglobulins using magnetic beads with the viral antigen immobilized on their surface (Fiordelisio et al., 2021).

Tests were carried out to determine the standardization parameters of the SARS-CoV-2 AbDM implemented both in a multiwell plate and in a microfluidic chip, such as the concentration of antigen (RBD or S protein) to functionalize the magnetic beads, the concentration of secondary antibody, the detection limit, and the proportion of the serum sample dilution to be used. Validation of the SARS-CoV-2 AbDM was performed with different samples, using ELISA as a reference method. Likewise, assays were performed with the SARS-CoV-2 AbDM for the detection of whole blood as a sample and the co-detection of both IgG and IgM in the same serum sample.

Based on the results obtained, the ELISA method appears to be more sensitive than the SARS-CoV-2 AbDM for both RBD and S protein (Figure 2C; Figure 3C; Figure 7B), reaching a plateau in absorbance at high serum protein concentration values. On the other hand, the SARS-CoV-2 AbDM did not reach saturation, which indicates that it has a greater range in its ability to determine higher values than the ELISA. In addition, it should be noted that, in the detection of antibodies by ELISA, the first step is to determine whether the sample is positive or negative, and then, a titration process is performed to determine the concentration of the sample within a range, which is dependent on the color generated by an enzymatic reaction. This process can easily vary depending on the time and temperature. In the case of SARS-CoV-2 AbDM, this variation does not exist since there is no such process, and the detection is performed directly.

Although the performance of the ELISA, determined by a dilution curve, shows high sensibility compared to the SARS-CoV-2 AbDM when used as a diagnosis with patient samples, the first value is that of a titer of 50 (0.4 OD), corresponding to 0.71 μg/μL. In this sense, the diagnosis by ELISA is not only done in two steps with six analyses that consume twice the time and supplies, but its first level of detection is also closer to that of the SARS-CoV-2 AbDM. Thus, the developed method would allow a quantitative measurement of the antibody concentration in a single assay with a higher dynamic range and a similar detection limit (2.13 μg/μL).

As for the comparison of the normalized fluorescence intensity obtained with SARS-CoV-2 AbDM in serum samples, an upward trend is observed with respect to the ELISA titers, which is the expected behavior, since the higher the ELISA titer, the higher the fluorescence intensity should be (Figures 4A, D). When using S protein as antigen, a better resolution was observed among the ELISA titers than with RBD, which coincides with the data obtained and previously discussed from the standardization process. It is evident that, for each ELISA titer, a great variability is reflected in the fluorescence intensity values since a limitation of the ELISA is that it groups the determinations in ranges, i.e., the absorbance value obtained is classified according to the last dilution of the sample that exceeds the cut-off point threshold. In contrast, with the SARS-CoV-2 AbDM, the fluorescence intensity value is continuous, thus giving a spectrum of fluorescence values for a single ELISA titer value, providing greater resolution.

Regarding the validation of the SARS-CoV-2 AbDM on a plate for the detection of anti-SARS-CoV-2 IgG, and based on the ROC analysis performed, we consider the specificity (RBD, 71%; S protein, 100%) and sensitivity (RBD, 84%; S protein, 100%) values to be excellent for S protein as antigen, RBD less so. This difference observed between RBD and S protein may be due to the inherent properties of these proteins; for example, since S protein is larger (1273 aa vs. 319-541 aa) and has more antibody binding sites than RBD, the stoichiometry with the antibody is more favorable. Therefore, the orientation of RBD in the bead attachment or on the reaction surface could compromise the exposure of the antibody binding site and, therefore, contributes to the variability observed with this protein as antigen. Nevertheless, the evaluation of specificity would benefit greatly from more negative samples, but we had no serum samples from before the pandemic began.

In common practice, in order to avoid high background signal, when making immunoglobulin determinations in blood by ELISA, serum samples are usually used instead of whole blood (Kovac et al., 2020); the main problem of the background is that it makes it difficult to differentiate low values. We demonstrated that, with the SARS-CoV-2 AbDM, the reaction is specific, and the reaction surface is clean to such a degree that we can use whole blood or serum without modifying the value obtained. This raises the possibility of developing a PoC-type device that uses whole blood as a sample, like the rapid LFIAs, but with a quantitative result and greater sensitivity and specificity. However, it is important to note that for this evaluation, three samples were used.

Another attribute of the SARS-CoV-2 AbDM is that, by using fluorescence as the marker signal instead of a colorimetric enzymatic reaction, such as ELISA, it can be used to perform multiple detections with the same sample. Thus, with the proposed method, we achieve the co-detection of IgG and IgM anti-SARS-COV-2 antibodies in a single sample simultaneously. This represents a great advantage over ELISA since it saves resources in terms of time and consumables by not needing to carry out independent determinations.

The versatility of the SARS-CoV-2 AbDM allowed it to be implemented for IgG-S detection on a microfluidic chip toward a PoC, with a motorized platform (Fiordelisio et al., 2021) that allowed for a precise reaction and manipulation of the samples up to the optical detection stage. As with the plate-based version of the method, an upward trend of fluorescence intensity with respect to the ELISA titers and variation in their respective ranges was observed on the chip, as expected. Furthermore, the specificity (100%) and sensitivity (94%) values after ROC analysis are adequate and promising for future development as a PoC device (Figure 7). In addition, another great advantage of using the SARS-CoV-2 AbDM on the microfluidic chip is that the operator only needs to place the sample in the well of the chip since the platform automates the reaction process up to the use of the detection device. This reduces the human error associated with handling and frees up the user’s time for other matters.

The cost of performing SARS-CoV-2 AbDM in American dollars was 6 USD for the 96-wells plate and microfluidic chip compared to the ELISA’s 13 USD cost. Since ELISA requires a series of dilutions in several wells to perform the titration of the samples, the space available on a single plate is limited to a few samples (approximately eight, including controls), so a strategy is usually followed, as the one we follow, where first an ELISA is performed only for screening of positives or negatives and then selecting only the positives to determine the titer (Stadlbauer et al., 2020; Martínez-Barnetche et al., 2022), thus doubling the time to 16 h, resources and cost invested. In contrast, with the SARS-CoV-2 AbDM, by not requiring serial dilutions, with a single sample in a reaction well, the result can be obtained in 5 h on a plate or 1.5 h on a chip, without the need for prior scanning, with the possibility of multiple detections.

Since the reaction surface of the SARS-CoV-2 AbDM does not depend on a fixed area but on the number of beads, an advantage of this method is the ability to modify the dynamic range; for example, by increasing the number of beads, the reaction surface area is increased, thus allowing more antibodies to be captured. Another possibility is to reduce the dilution of the serum sample, which increases the number of antibodies available for detection, resulting in a higher detection signal. All these are opportunities to settle parameters depending on antibodies and antigens used to measure.

In short, we have presented a quantitative method with similar sensitivity to an ELISA, in less time, and with a cost comparable to that of a LFIA test, with a high potential to be developed as a PoC and thus be an alternative tool for monitoring seroprevalence of SARS-CoV-2 antibodies (Table 1). Both the present work and the one previously reported by our group (Fiordelisio et al., 2021) demonstrate the versatility of the method based on the bead system as a reaction surface and allow us to believe in a wide range of applications in other biomolecules.

**TABLE 1:** Comparison of the main serological detection methods for antibodies against SARS-CoV-2.

	Fast test	ELISA	SARS-CoV-2 AbDM
Sensitivity	54.3%–99%	65%–100%	84%–100%^a^
Specificity	94.1%–100%	84.4%–100%	71%–100%^b^
Cost	10.22–28.33 USD^✝^	53.87–113.54 USD^✝^	6 USD
Simplicity	Capillary blood auto test	Trained personnel and requires multiple assays	Automated with chip
Time for result	10–20 min	16 h	1.5 h
Response type	Binary +/−	Semi-quantitative	Quantitative
Co-detection	Yes	No	Yes
Sample type	Blood	Serum	Blood/serum

^a^ Sensitivity for RBD in plate is 84%, S protein in plate is 100%, and S protein on chip is 94%.

^b^ Specificity for RBD in plate is 81%, S protein in plate is 100%, and S protein on chip is 100%.

^✝^ The costs of the tests are from the main clinical laboratories in Mexico.

Adapted from Gong et al.

The SARS-CoV-2 AbDM designed to detect IgG and IgM antibodies against SARS-CoV-2 S protein and RBD in human serum samples (assayed in well plates and microfluidic chip) proved to be comparable to conventional ELISA assays, especially for S protein as antigen. The SARS-CoV-2 AbDM provided several advantages in testing, such as cost reduction, the possibility of performing single or multiplex assays, the possibility of multiplex detection, and the ability to detect whole blood samples without loss of resolution. In addition, with the microfluidic chip in conjunction with the motorized actuated platform, there is the advantage of automation, reduction of time, sample quantity, and operator intervention during the process. All these advantages suggest that the SARS-CoV-2 AbDM can be used as a tool for seroprevalence monitoring, allowing a better understanding of the epidemiological and clinical characteristics of COVID-19 and contributing to more effective and ethical decision-making in strategies to combat the COVID-19 pandemic. The demonstrated versatility of the method and its eventual implementation in PoC made it possible to offer greater availability of analytical tests for other biomolecules, whether of health or non-health interest, in places where the use of expensive and complex instruments is not an option.

## Data Availability

The original contributions presented in the study are included in the article/supplementary material, further inquiries can be directed to the corresponding author/s.
